# Oxalic Acid in Rhubarb or Pie Plant

**Published:** 1845-06-01

**Authors:** 


OXALIC ACID IN RHUBARB OR PIE PLANT.—A family of four persons, in this city, recently, after eating freely of the leaves of the domestic Rhubarb or Pie plant, boiled, and served as "greens," were, all of them, shortly seized with severe vomiting. In one of the persons it was followed by gastritis. The others recovered directly after the vomiting. We have occasionally seen notices in newspapers of this plant producing noxious effects. Will some one of our chemical friends furnish us with a quantitative analysis of it, with reference especially to the proportion of Oxalic Acid which it contains?

